# Identifying the Source of a Humoral Factor of Remote (Pre)Conditioning Cardioprotection

**DOI:** 10.1371/journal.pone.0150108

**Published:** 2016-02-26

**Authors:** Svetlana Mastitskaya, Marina Basalay, Patrick S. Hosford, Andrew G. Ramage, Andrey Gourine, Alexander V. Gourine

**Affiliations:** 1 Centre for Cardiovascular and Metabolic Neuroscience, Neuroscience, Physiology and Pharmacology, University College London (UCL), London, United Kingdom; 2 Department of Cardiology, Karolinska University Hospital, Stockholm, Sweden; University of Louisville, UNITED STATES

## Abstract

Signalling pathways underlying the phenomenon of remote ischaemic preconditioning (RPc) cardioprotection are not completely understood. The existing evidence agrees that intact sensory innervation of the remote tissue/organ is required for the release into the systemic circulation of preconditioning factor(s) capable of protecting a transplanted or isolated heart. However, the source and molecular identities of these factors remain unknown. Since the efficacy of RPc cardioprotection is critically dependent upon vagal activity and muscarinic mechanisms, we hypothesized that the humoral RPc factor is produced by the internal organ(s), which receive rich parasympathetic innervation. In a rat model of myocardial ischaemia/reperfusion injury we determined the efficacy of limb RPc in establishing cardioprotection after denervation of various visceral organs by sectioning celiac, hepatic, anterior and posterior gastric branches of the vagus nerve. Electrical stimulation was applied to individually sectioned branches to determine whether enhanced vagal input to a particular target area is sufficient to establish cardioprotection. It was found that RPc cardioprotection is abolished in conditions of either total subdiaphragmatic vagotomy, gastric vagotomy or sectioning of the posterior gastric branch. The efficacy of RPc cardioprotection was preserved when hepatic, celiac or anterior gastric vagal branches were cut. In the absence of remote ischaemia/reperfusion, electrical stimulation of the posterior gastric branch reduced infarct size, mimicking the effect of RPc. These data suggest that the circulating factor (or factors) of RPc are produced and released into the systemic circulation by the visceral organ(s) innervated by the posterior gastric branch of the vagus nerve.

## Introduction

Remote ischaemic preconditioning (RPc) cardioprotection is the phenomenon whereby cycles of ischaemia/reperfusion applied to an organ or tissue remote from the heart protect cardiomyocytes against lethal ischaemia/reperfusion injury [1]. Despite recent advances in understanding the mechanisms underlying RPc cardioprotection (since first described by Przyklenk *et al*. in 1993 [2]) and promising results of some human trials in translating RPc into clinical practice, the signalling mechanisms of RPc remain incompletely understood [3, 4]. In particular, it is not entirely clear how the “protective stimulus” is communicated from the remote organ to the heart. Several studies suggested the roles of both humoral (i.e. circulating protective factor(s)) [5–8] and neural [1, 9–12] signalling pathways.

The majority of the existing evidence agrees that intact afferent (sensory) innervation of the remote tissue/organ is required to mediate cardioprotection established by a variety of peripherally-applied stimuli, including “classical” RPc involving episodes of remote ischaemia/reperfusion [9, 10], topical application of capsaicin to activate sensory fibres [8] or electroacupuncture [13]. Intact sensory innervation of the remote organ is also required for the production of transferable “remote preconditioning factor” capable of protecting denervated or transplanted heart [8, 14]. Efficacy of this circulating factor was also demonstrated in a cross species RPc model, where plasma dialysate from human donors who underwent arm RPc was applied to a Langendorff rabbit heart preparation or cardiomyocyte cultures [6]. These data suggested that activation of sensory fibres, which innervate the remote tissue, results in a release of a preconditioning factor into the systemic circulation.

There is also strong evidence suggesting that intact parasympathetic mechanisms are required to establish RPc cardioprotection. It was shown that RPc cardioprotection is abolished by highly selective genetic targeting and silencing of vagal preganglionic neurons of the dorsal vagal motor nucleus (DVMN), bilateral cervical vagotomy or systemic muscarinic receptor blockade with atropine [9, 11, 15–17]. We and others [18–20] have demonstrated significant vagal innervation of the left ventricle and suggested that RPc is mediated by the actions of acetylcholine released by vagal efferents at the level of the myocardium, completing the arch of the “remote preconditioning reflex” [11, 16, 21].

However, the significance of parasympathetic ventricular innervation has been questioned previously (reviewed by J Coote [22]) and the proposed vagal mechanism cannot fully explain the efficacy of RPc in protecting denervated or transplanted hearts or cross-species transfer of ‘cardioprotection’ by plasma dialysate. Overall, the data showing a critical role of vagal mechanisms in mediating RPc phenomenon suggested that release of a transferable RPc factor is under parasympathetic control mediated by muscarinic receptor activation. Results of recent reports of the proteomic analysis of the RPc dyalisate identified changes in plasma levels of certain proteins [23]; some of these are produced by the liver.

In this study we tested the hypothesis that RPc stimulus applied to the peripheral organ/tissue triggers release of a preconditioning factor (or factors) from visceral organs, which receive rich vagal innervation. This was carried out by determining if the efficacy of RPc in establishing cardioprotection is affected by subdiaphragmatic vagotomy and by sectioning of individual branches of the abdominal vagus nerve: celiac, hepatic, anterior gastric and posterior gastric. Electrical stimulation of the peripheral end of the individually sectioned vagal branches was then carried out to determine whether enhanced vagal input to a particular visceral target area was sufficient to establish (i.e. mimic RPc) cardioprotection.

## Materials and Methods

All the experiments were performed in accordance with the European Commission Directive 2010/63/EU (European Convention for the Protection of Vertebrate Animals used for Experimental and Other Scientific Purposes) and the UK Home Office (Scientific Procedures) Act (1986) with the project approval from the UCL Animal Welfare and Ethical Review Body (AWERB).

### Animal preparation

This study was performed in 69 adult male Sprague-Dawley rats (280–320 g; UCL breeding colony) anaesthetized with pentobarbital (induction 60 mg kg^−1^ i.p.; maintenance 10–15 mg kg^−1^ h^−1^ i.v.). Adequate anaesthesia was ensured by maintaining stable levels of the arterial blood pressure (ABP) and heart rate and monitored by the absence of a withdrawal response to a paw pinch. The right carotid artery and left jugular vein were cannulated for measurement of ABP and administration of anaesthetic, respectively. The trachea was cannulated, and the animal was mechanically ventilated with room air using a positive pressure ventilator (tidal volume of 1 ml/100 g of body weight, ventilator frequency ∼60 strokes min^−1^). *P*O_2_, *P*CO_2_ and pH of the arterial blood were measured regularly and, if required, ventilation was adjusted to maintain these values within the physiological ranges. ABP and a standard lead II ECG were recorded using Power1401 and *Spike2* software (Cambridge Electronic Design). The body temperature was maintained at 37±0.5°C with a servo-controlled heating pad.

### Subdiaphragmatic and selective visceral vagotomies

**Fig 1:**
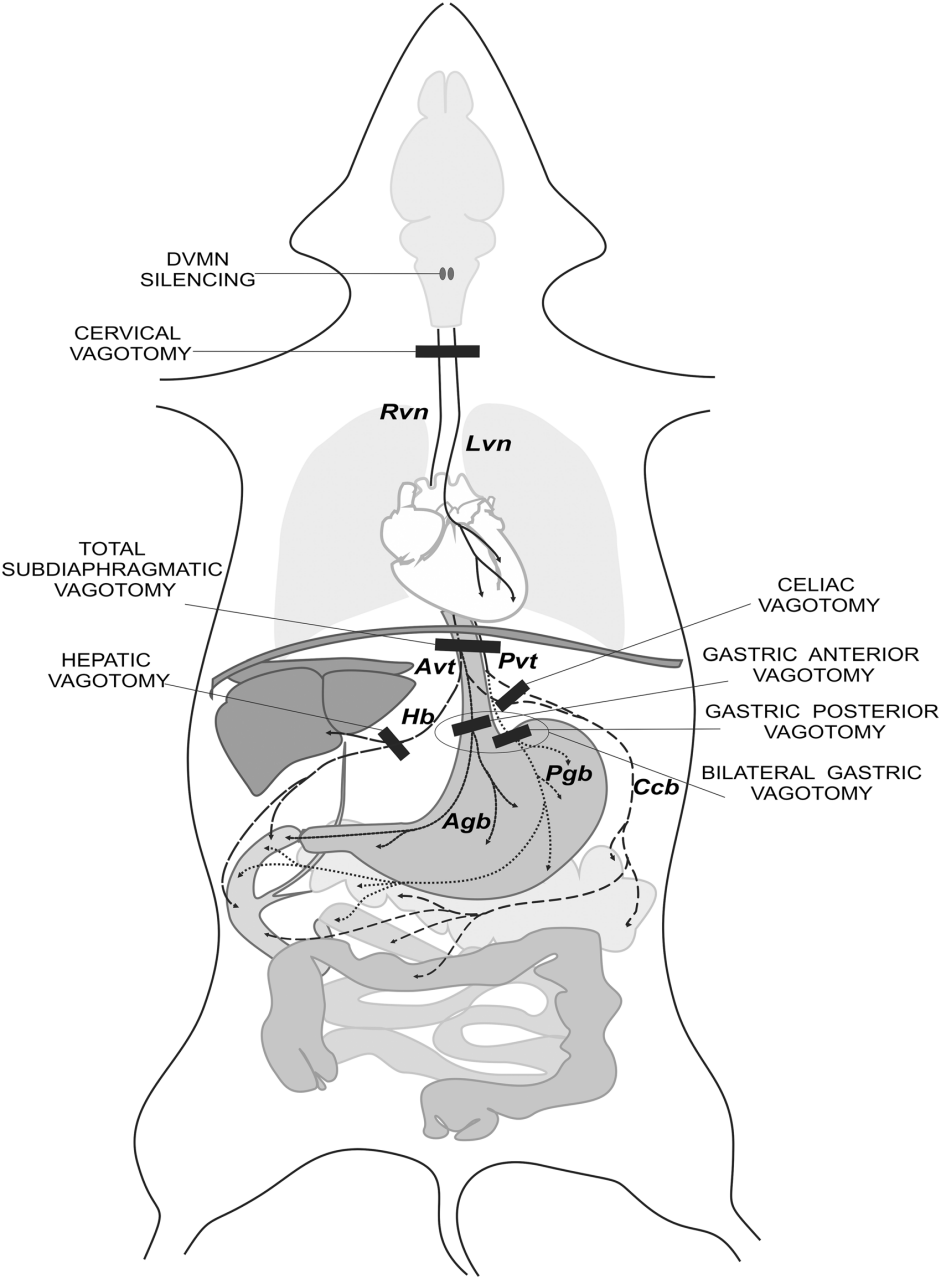
Experimental interventions. Previous studies—DVMN silencing, cervical vagotomy. Six types of subdiaphragmatic vagotomy performed in the current study: total, bilateral gastric, anterior gastric, posterior gastric, hepatic and celiac, are shown on a schematic representation of typical distribution of rat abdominal vagal branches. Agb, anterior gastric branch; Avt, anterior vagal trunk; Ccb, common celiac branch; Hb, hepatic branch; Lvn, left vagus nerve; Pgb, posterior gastric branch; Pvt, posterior vagal trunk; Rvn, right vagus nerve. Brain, lungs, heart, diaphragm, liver, stomach, pancreas, small intestine and colon are depicted schematically.

Vagal trunks were carefully dissected bilaterally under the diaphragm as described by Prechtl *et al*. [24]. Briefly, an incision was made to gain access to the abdominal cavity. The left lobes of the liver were gently pulled aside and the stomach was retracted caudally to expose the esophagus. Subdiaphragmatic vagal trunks or individual vagal branches were identified and isolated. Experimental groups were formed from the animals which had a total subdiaphragmatic vagotomy (i); and those which had individual branches sectioned, the hepatic branch (ii); the celiac branch (iii); the gastric posterior branch, (iv), the gastric anterior branch (v) and both gastric branches (vi) (Fig 1). In rats receiving sham treatment the preparative surgery and all the procedures were identical, vagal branches were visualized, subjected to gentle traction applied to their fascial investiture but not sectioned. The stomach and the lobes of the liver were then repositioned back within the abdominal cavity and the abdominal muscle and skin were closed with surgical sutures. RPc or sham-RPc stimuli were applied 15 min after the vagotomies.

### Electrical stimulation of individual vagal branches

Individual visceral branches of the vagus nerve were isolated as described above. Care was taken when separating the nerves so as not to damage others within the bundle. After isolation, the branch was cleared of connective tissue, the proximal end was ligated with a silk suture and crushed to prevent afferent traffic. The nerve was then placed on a bipolar silver hook stimulating electrode with the cathode positioned proximally to the target organ. The electrode was connected to a constant current isolated stimulator (Model DS3; Digitimer) triggered by a digital output from Power1401 interface controlled by a script written for *Spike2* software. The exposed nerve (free and placed on the stimulating electrodes) was embedded in polyvinylsiloxane dental impression material (Super-Dent^®^, Carlisle Laboratories). The stomach and the lobes of the liver were then repositioned back within the abdominal cavity and the abdominal muscle and skin were closed with surgical sutures. The electrical stimulation (10 Hz, 0.5 mA, 0.1 ms pulse) was delivered continuously starting 25 min prior to the left anterior descending (LAD) artery ligation and continuing 10 min into the reperfusion period, in accord with the protocol described previously [11]. Parameters of electrical stimulation were similar to those used in studies of vagal efferent control of gastric and intestine motility [25]. In animals receiving sham stimulations, the surgical procedures were identical, the nerve was crushed and placed on electrodes but no current was delivered to the electrodes.

### Induction of RPc cardioprotection

An established experimental model of RPc cardioprotection was used [9, 11]. Blood supply to both hind limbs was interrupted for 15 min by placing vessel clamps on both femoral arteries at the proximal level ∼1 cm below the inguinal ligament. The clamps were removed and the limbs perfusion was reinstated for 10 min prior to myocardial infarction. Sham-RPc procedure involved dissection of femoral arteries without occlusion. This RPc protocol (15 min occlusion of both femoral arteries followed by 10 min of reperfusion) was previously reported to confer significant cardioprotection against acute myocardial ischaemia/reperfusion injury [9, 11, 26].

### Myocardial ischaemia/reperfusion injury

The heart was exposed via a left thoracotomy and a 4–0 monofilament polypropylene suture was passed around the LAD coronary artery to induce a temporary occlusion. The animals were subjected to 30 min of LAD artery ligation, followed by 120 min of reperfusion. Successful LAD occlusion was confirmed by elevation of the ST-segment in the ECG and an immediate 15–30 mmHg fall in the ABP.

### Measurements of infarct size

At the end of the reperfusion period, the LAD artery was re-occluded and 1 mL of 1.5% Evans blue dye was injected into the jugular vein to determine the area at risk. The animal was then given an anaesthetic overdose (pentobarbital sodium 200 mg kg^−1^ iv), the heart was excised, left ventricle was isolated, frozen, and sectioned into 5–6 transverse slices from the apex to the base. The slices were weighed and photographed. The area at risk was demarcated by the absence of Evans blue staining. The slices were then incubated in 1% solution of 2,3,5- triphenyltetrazolium chloride (TTC) in Tris buffer (pH 7.4) for 15 min at 37°C, fixed in 4% formalin for 24 h, and photographed again. Viable myocardium is stained red by TTC, whereas necrotic myocardium appears pale yellow. The area at risk and the necrotic area were determined by computerized planimetry, normalized to the weight of each slice, with degree of necrosis (i.e. infarct size) expressed as the percentage of area at risk.

### Statistical analysis

Data are reported as individual values and means ± SEM. Groups were compared by Kruskal–Wallis ANOVA by ranks followed by the Dunn’s post-hoc tests. Values of p< 0.05 were considered to be significant.

## Results

**Fig 2:**
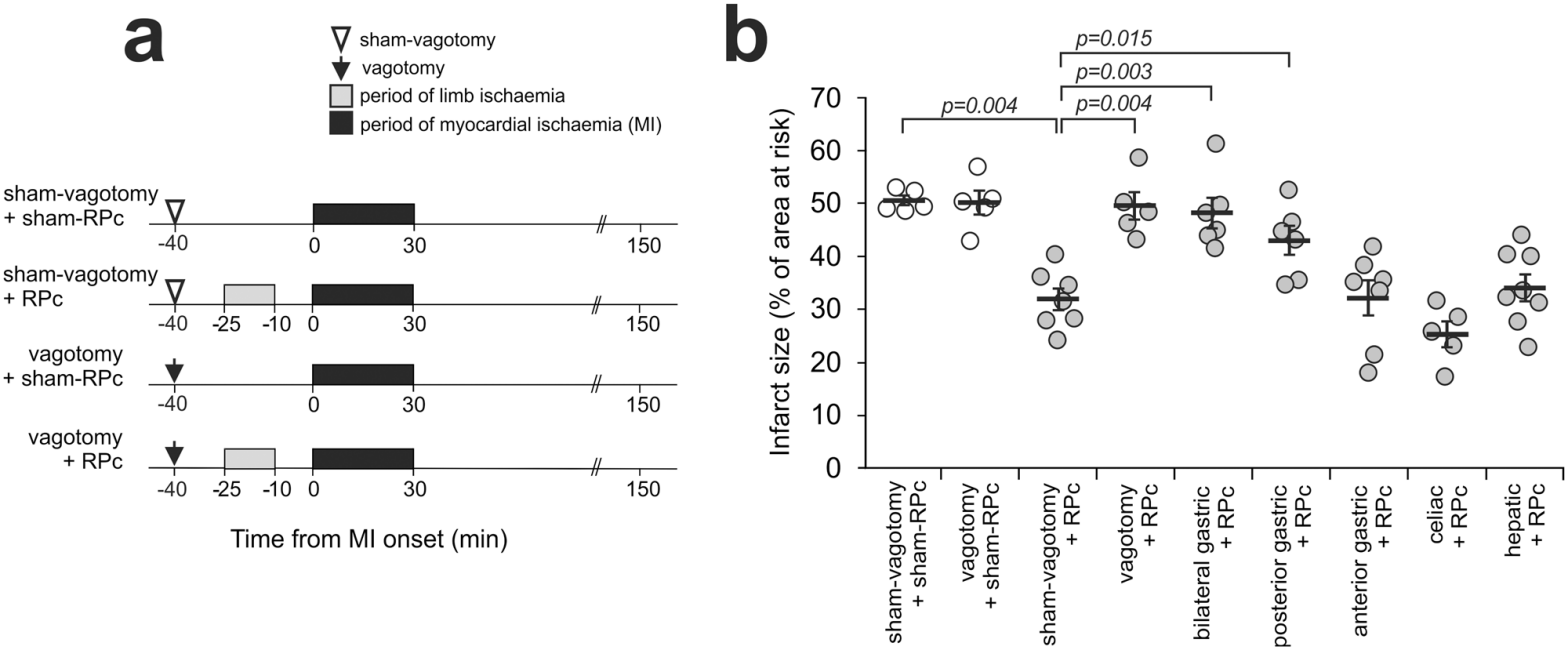
Cardioprotection established by remote ischaemic preconditioning (RPc) requires intact parasympathetic innervation of visceral organs. **(a)** Illustration of the experimental protocols. RPc was induced by 15 min occlusion of both femoral arteries, followed by 10 min reperfusion. Sham-RPc procedure involved dissection of both femoral arteries without occlusion. Arrows indicate time of total subdiaphragmatic vagotomy, selective sectioning of individual visceral branches or sham surgery. **(b)** Total subdiaphragmatic vagotomy, bilateral gastric vagotomy and selective sectioning of the posterior gastric branch abolished the cardioprotective effect of RPc, whereas sectioning of the anterior gastric, celiac or hepatic branches had no effect on RPc cardioprotection. The infarct size is presented as the percentage of the area at risk. Individual data and means ± SEM are shown. P-values correspond to the Dunn’s post-hoc tests.

**Fig 3:**
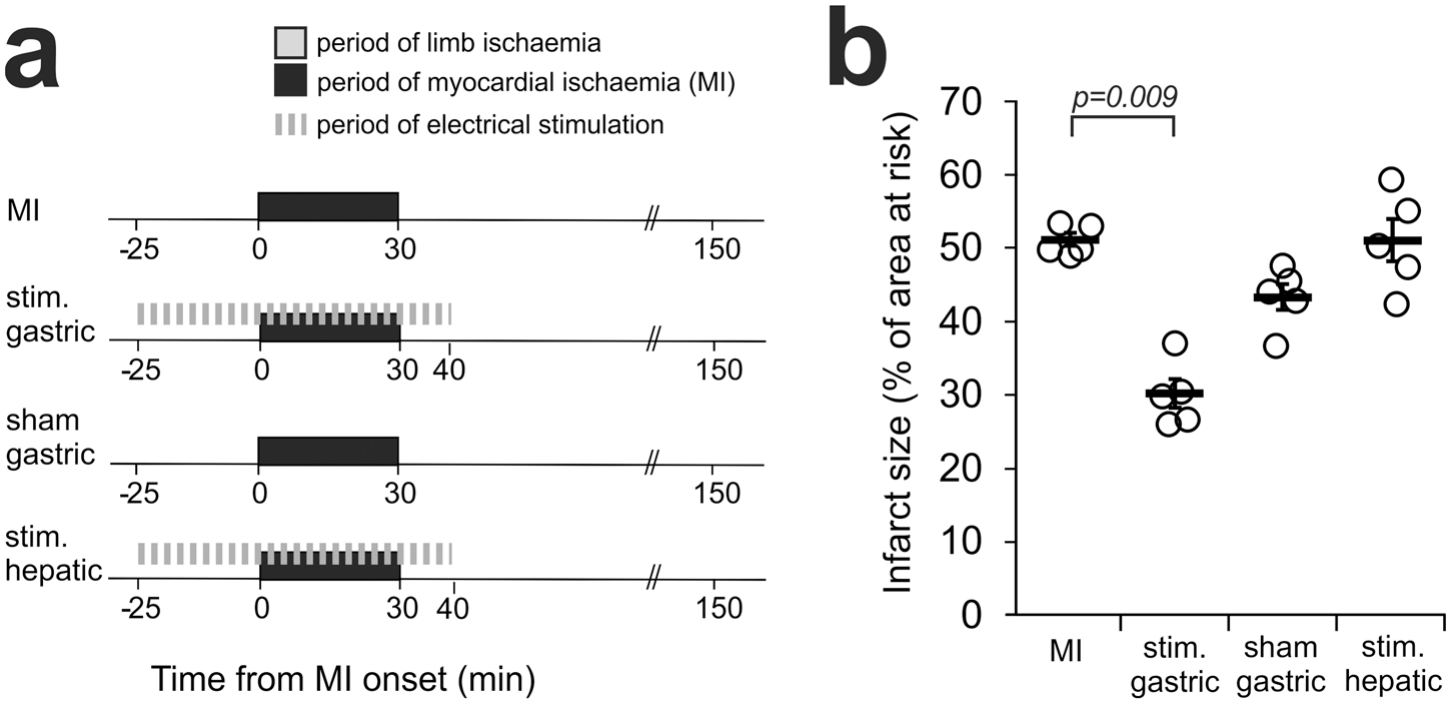
Electrical stimulation of the posterior gastric vagal branch mimics RPc cardioprotection. **(a)** Illustration of the experimental protocols. Electrical stimulation (stim.) of individual vagal branches commenced 25 min before the onset of myocardial ischaemia (MI) and continued 10 min into the period of reperfusion. Sham procedure involved surgical dissection of the nerve and placing it on the electrodes without stimulation. **(b)** Electrical stimulation of the posterior gastric vagal branch reduced the extent of myocardial ischaemia/reperfusion injury, whereas stimulation of the hepatic vagal branch or sham stimulation of the posterior gastric branch had no effect. The infarct size is presented as the percentage of area at risk. Individual data and means ± SEM are shown. P-values correspond to the Dunn’s post-hoc tests.

No differences in mean ABP or heart rate before or during ischaemia and reperfusion were observed between groups of animals recruited into the experimental protocols (S1 Table). There were also no differences in the areas at risk between the experimental groups. In our experiments no reduction in infarct size was observed in animals receiving abdominal incision (50±1% in a control group vs 53±2% in a group receiving laparotomy, data not shown). Surgical access to the abdominal cavity was identical in all the experiments. Figs 2 and 3 illustrate infarct size data displayed as percentages of the area at risk.

Thirty min of myocardial ischaemia (LAD occlusion) followed by 120 min of reperfusion resulted in a mean infarct size of 53±2% (Fig 2). Significant cardioprotection was established by application of the RPc stimulus (15 min occlusion of femoral arteries followed by 10 min of reperfusion) as evident from a significant reduction in infarct size (32±2%, p = 0.004; Fig 2). Total subdiaphragmatic vagotomy abolished RPc cardioprotection (infarct size 50±3%; p = 0.004) and had no effect on the infarct size in animals not subjected to RPc (50±2%) (Fig 2). These data suggested that RPc cardioprotection requires intact vagal innervation of the visceral organs.

Experiments involving selective cuts of individual vagal branches demonstrated that the efficacy of RPc cardioprotection is preserved in conditions of hepatic, celiac or anterior gastric vagotomy (infarct sizes 34±3%, 26±2% and 32±3%, respectively; Fig 2). Sectioning of both anterior and posterior gastric branches or posterior gastric branch only abolished RPc cardioprotection (infarct sizes 49±3% and 43±3%, p = 0.003 and p = 0.015, respectively, Fig 2).

These data suggest that the RPc humoral factor(s) is likely to be produced (and released into the systemic circulation) by the visceral organ(s) innervated by the posterior gastric branch of the vagus nerve. By extension, electrical stimulation of this branch in the absence of RPc stimulus should result in cardioprotection. It was found that electrical stimulation of the posterior gastric branch established cardioprotection, mimicking the effect of RPc (infarct size 30±2% vs 53±2% in control animals, p = 0.009; Fig 3). Neither sham stimulation of the posterior gastric branch nor electrical stimulation of the hepatic branch had an effect on the extent of the myocardial ischemia/reperfusion injury (43±2 and 51±3%, respectively; Fig 3).

## Discussion

There is significant evidence suggesting that the mechanisms of RPc cardioprotection involve circulating humoral factor(s) produced during ischemia/reperfusion of the remote tissue [6, 10, 27–30], a neural component [1, 9–12, 14, 31–33], or both [8, 10]. Unifying current view of the general signalling mechanism of RPc involves afferent (sensory) innervation of the remote organ, which is required for the production and release into the systemic circulation of a transferable preconditioning factor [7, 8].

There is also significant evidence demonstrating that RPc is only effective in establishing cardioprotection if vagal mechanisms are intact. Experimental data obtained in our laboratory and by other groups showed that RPc cardioprotection is abolished in conditions of bilateral cervical vagotomy [9, 15, 17], systemic muscarinic receptor blockade [9, 15, 17] or selective genetic targeting and silencing of brainstem vagal preganglionic neurons [11]. Earlier we proposed the idea of a “remote preconditioning reflex” which suggested that activation of C-fiber afferents (by ischaemia/reperfusion or noxious stimuli) innervating the remote tissue/organ is leading to an increased activity of parasympathetic fibers innervating the ventricular myocardium which then establishes cardioprotection via release and actions of acetylcholine [11]. However, RPc is also effective in protecting denervated or transplanted hearts [27] and plasma dialysate obtained from humans receiving RPc stimulus is effective in protecting the recipient (rabbit) heart (Langendorff preparation) against ischaemia/reperfusion injury [32] arguing against a critical role of direct vagal innervation of the ventricle. Interestingly, the same study demonstrated that plasma dialysates obtained from diabetic patients with peripheral neuropathy (i.e. parasympathetic dysfunction) were not effective [32].

Analysis of the data available in the literature allowed us to hypothesize that production and release of a circulating preconditioning factor is under parasympathetic control. As inhibition of DVMN vagal preganglionic neurons abolishes RPc cardioprotection [11] and the majority of DVMN neurons project to the myenteric plexus, with the highest density of efferent fibers terminating in the stomach [25], we determined the efficacy of RPc cardioprotection first in conditions of total subdiaphragmatic vagotomy and then following selective parasympathetic denervation of various visceral targets. The data obtained suggest that the circulating RPc factor is released by the internal organs innervated by the posterior gastric branch of the vagus nerve, which include stomach, proximal duodenum, jejunum and parts of the pancreas [25].

In an attempt to identify the nature of the preconditioning factor(s) Lang et al. [34] conducted a proteomic analysis on blood samples obtained from experimental animals following application of the RPc stimulus. Results of that study did not support the existence of a circulating cardioprotective factor with a molecular weight of more than 8 kDa [34]. More recent study conducted in healthy human volunteers reported that RPc is associated with differential regulation of several plasma proteins linked to the control of an acute phase response and various cellular functions [23]. Among potential humoral factors identified in plasma-derived dialysate only three were reported to have a cardioprotective effect: nitrite [35], microRNA-144 [36] and stromal derived factor-1α [37]. Their actions, however, may not fully explain the RPc phenomenon, especially in light of a recent clinical report showing that nitrite infusion fails to establish cardioprotection against myocardial injury in humans [38].

Various experimental pre-clinical models (the majority of them involving young or very young healthy animals) demonstrated that RPc is highly effective in protecting the heart against lethal ischaemia/reperfusion injury. Clinical trials designed to determine the efficacy or remote (pre)conditioning in establishing cardioprotection yielded both positive [39–41] and neutral results [42–45]. In view of the results obtained in the present study, the neutral outcomes of the most recent trials ERICCA [44] and RIPHeart [45] are not surprising. In both trials patients received propofol, an anesthetic agent known to suppress the activity of vagal preganglionic neurons and inhibit autonomic reflex pathways [46, 47]. Moreover, parasympathetic tone decreases with age and could be severely diminished or even absent in many disease states, perhaps rendering many patients of an advanced age (recruited in these trials) insensitive to this procedure. Either the heart may no longer be able to sense increased level of circulating remote preconditioning factor or parasympathetic dysfunction results in a compromised ability of certain groups of patients to produce the preconditioning factor. The data obtained in diabetic patients with peripheral neuropathy [32] provide strong evidence in favor of the latter.

**Fig 4:**
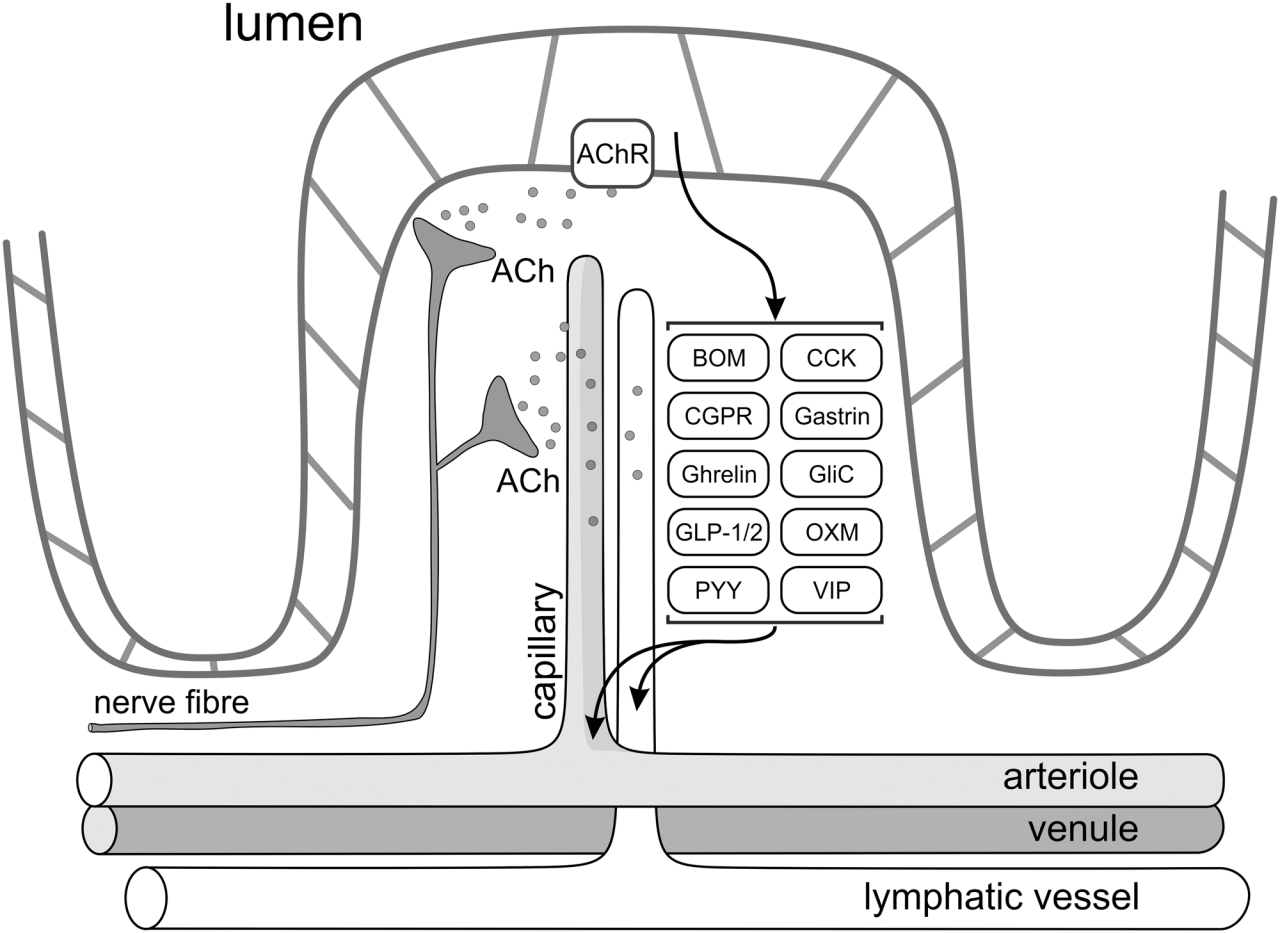
Diagrammatic representation of the nervous control of hormone secretion by enteroendocrine cells of the gastrointestinal tract. Extrinsic vagal parasympathetic nerves either directly or via activation of the enteric neurones trigger release of hormones (hypothesised circulating cardioprotective factors) by releasing acetylcholine (among other transmitters). ACh, acetylcholine; AChR, acetylcholine receptor; BOM, bombesin; CCK, cholecystokinin; CGPR, calcitonin gene-related peptide; GliC, glicentin; GLP-1/2, glucagon-like peptide-1 and 2; OXM, oxyntomodulin; PYY, peptide YY; VIP, vasoactive intestinal peptide.

Identifying the nature of circulating remote preconditioning factor(s) is, therefore, important as it would allow its potential application as a mainstream cardioprotective strategy in a clinical setting. This study represents a significant step forward in a quest to reveal the identity of this factor which appears to be produced and released by the visceral organs innervated by the posterior branch of the vagus nerve. Certain gut hormones released into the circulation in response to enhanced vagal activity might as well fulfil this important role (Fig 4).

## Supporting Information

S1 Table Haemodynamic Data. (PDF)
