# Supplementary material for: Identifying the Source of a Humoral Factor of Remote (Pre)Conditioning Cardioprotection
Source: PLoS One. 2016 Feb 26;11(2):e0150108. doi: 10.1371/journal.pone.0150108 (PMC4769182; doi:10.1371/journal.pone.0150108)
Supplement: S1 Table — (PDF) [file pone.0150108.s001.pdf]

S1 Table. Haemodynamic data.

					Reperfusion (min)	
	**Variable**	**Basal**	**Pre ischemia**	**End ischemia**	**60**	**120**
Subdiaphragmatic vagotomy						
Sham-RPc (n=5)	MAP	101 ± 6	83 ± 5	85 ± 4	82 ± 4	74 ± 4
	HR	458 ± 15	467 ± 18	469 ± 17	426 ± 11	409 ± 15
RPc (n=7)	MAP	115 ± 5	91± 5	75 ± 8	89 ± 5	72 ± 7
	HR	477 ± 14	480 ± 17	478 ± 11	438 ± 12	425 ± 19
Vagotomy + sham RPc (n=5)	MAP	101 ± 6	92 ± 13	81 ± 13	88 ± 14	84 ± 13
	HR	451 ± 19	464 ± 15	458 ± 15	431 ± 21	401 ± 17
Vagotomy + RPc (n=5)	MAP	102 ± 5	87 ± 5	65 ± 5	75 ± 7	65 ± 7
	HR	475 ± 18	485 ± 19	500 ± 16	474 ± 12	464 ± 16
Hepatic + RPc (n=8)	MAP	99 ± 7	75 ± 7	72 ± 8	69 ± 10	65 ± 9
	HR	452 ± 15	441 ± 15	458 ± 16	412 ± 14	405 ± 19
Celiac + RPc (n=5)	MAP	108 ± 3	103 ± 4	82 ± 7	75 ± 7	69 ± 9
	HR	457 ± 16	476 ± 15	486 ± 11	471 ± 16	479 ± 19
Anterior gastric +RPc (n=7)	MAP	102 ± 8	95 ± 5	78 ± 5	72 ± 6	60 ± 6
	HR	478 ± 16	486 ± 21	496 ± 11	449 ± 14	431 ± 9
Posterior gastric + RPc (n=6)	MAP	105 ± 4	95 ± 5	72 ± 6	76 ± 7	73 ± 6
	HR	456 ± 17	471 ± 17	475 ± 18	471 ± 19	468 ± 18
Both gastric (n=6)	MAP	106 ± 5	85 ± 5	73 ± 4	77 ± 8	61 ± 7
	HR	454 ± 10	499 ± 10	481 ± 13	468 ± 11	443 ± 12
Electrical stimulation						
Gastric (n=5)	MAP	117 ± 8	110 ± 7	90 ± 7	85 ± 9	64 ± 6
	HR	454 ± 24	464 ± 14	468 ± 22	440 ± 15	423 ± 11
Gastric sham (n=5)	MAP	99 ± 6	81 ± 9	71 ± 10	73 ± 14	66 ± 13
	HR	437 ± 15	432 ± 23	447 ± 17	419 ± 18	435 ± 18
Hepatic (n=5)	MAP	101 ± 7	89 ± 6	89 ± 5	90 ± 4	72 ±8
	HR	428 ± 15	419 ± 14	457 ± 21	465 ± 24	478 ± 25
